# A Coarse-Grained Approach to Protein Design: Learning from Design to Understand Folding

**DOI:** 10.1371/journal.pone.0020853

**Published:** 2011-07-01

**Authors:** Ivan Coluzza

**Affiliations:** Department of Physics, University of Vienna, Vienna, Austria; National Institute for Medical Research, Medical Research Council, London, United Kingdom

## Abstract

Computational studies have given a great contribution in building our current understanding of the complex behavior of protein molecules; nevertheless, a complete characterization of their free energy landscape still represents a major challenge. Here, we introduce a new coarse-grained approach that allows for an extensive sampling of the conformational space of a large number of sequences. We explicitly discuss its application in protein design, and by studying four representative proteins, we show that the method generates sequences with a relatively smooth free energy surface directed towards the target structures.

## Introduction

Protein molecules play a central role in the large majority of biochemical reactions in living organisms [1]. Performance of these functions generally requires folding of the proteins into a specific three-dimensional structure, the so-called native state [2], [3], (a number of exceptions involving the so-called “disordered” proteins has also been discovered [4]). Computer simulations combined with experiments have given a great contribution to our current understanding of the complex behavior of protein molecules and of the mechanism by which folding takes place [3], [5], [6]. Advances have been made through the use of atomistic models, which are capable of providing detailed descriptions of protein dynamics [7], [5], [6], and through the development of coarse-grained representations, which enable more comprehensive sampling of the conformational space [3], [8], [9], [10].

A common approach to protein folding involves the use of Go-models [11]. The Go-models are non-transferable potentials tailored to the native structure such that each amino acid interacts selectively with a subset of residues and only when in the native configuration. Hence, Go-proteins are hypothetical proteins with a arbitrary variety of pair interactions among the residues (alphabet), but are able to successfully fold, and have a smooth free-energy landscape with a single global minimum in the native structure. However, if the size of the alphabet is reduced, for instance, to the 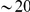 letter alphabet of real proteins, it becomes more and more difficult to observe folding for a random sequence, as the landscape most of the time changes from the smoothnes of Go-models, to rugged with many local minima. Hence, folding becomes more complex and requires an extensive search in the space of possible sequences to obtain a folding chain. For this reason these methods are often referred as “protein design”. Protein design was originally developed for lattice heteropolymers by Wolynes [12], and recently has been extended by Coluzza et al. [13]. By using lattice models it was possible not only to design heteropolymers with a large variety of target configurations, but also to generate lattice proteins with more complex self-assembly properties [14], [15]. The solution of the design problem is of considerable interest in biotechnology as it holds promises for the engineering of proteins with new functional properties. Some successful designs of novel artificial enzymes have been obtained by introducing residues expected to play a catalytic role in a specific reaction [16] in sequences with known folds.

In this work we will go beyond lattice models by introducing a novel design procedure that can produce realistic amino acid sequences able to fold into protein structures taken directly from experimental data. In what follows we will demonstrate for the first time that accurate representation of the protein backbone is a necessary condition for successful protein design, as such constraints confine the possible configurations of proteins to the structural space of real proteins. Our hypothesis is based on the observation that the design procedure developed for lattice proteins was unable to produce folding sequences when applied to simple off-lattice representations (e.g. a flexible chains of particles) (as indicated by our earlier simualtions). In order to understand the importance of constraints, let us ignore for a moment long range correlations in the system (i. e., we make a mean field approximation). Hence, energy minimization can be viewed as a local optimization of the residue-residue pair-interactions. In such condition, sequence mutation (design) is guaranteed to find the same minimum as configuration changes (folding) [12], provided that the number of possible sequences is larger or equal to the number of all possible configurations. Hence, for real proteins with an alphabet limited to 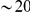 letters, it becomes clear that one needs to introduce constraints that limit the size of the configurational space (e.g. cubic lattice). Of course, in order to reproduce the space of real proteins, a specific set of constraints is needed that, contrary to Go potentials [11], does not vary from protein to protein.

Recently, Maritan and co-workers [17], [18], [19], have introduced a novel protein coarse-graining procedure by representing a typical protein as a flexible self-avoiding tube (from here the name “Tube” model) with a radius of 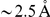 and effective hydrogen bonds interactions along the tube. The configurations of the tube model are controlled by just two parameters, the total hydrophobicity and the bending rigidity, that drive the tube into all secondary and many known protein’s tertiary structures. Hence, the results obtained with the tube model, strongly suggest that the typical protein structures are inherent in the geometrical constraints of the backbone, as the latter are the main features of the the tube model. To put in the words of the authors the tube “pre-sculpts” the free energy landscape. So far, a design method for the tube model has not been introduced and when hydrophilic/hydrophobic patterns of typical proteins were tried, the tube model could not systematically fold to the native structures [20]. However, we believe that the tube model highlighted the important type of constraints necessary to design sequences for real protein structure, namely the self-avoidance of the backbone and the hydrogen bonds.

**Figure 1:**
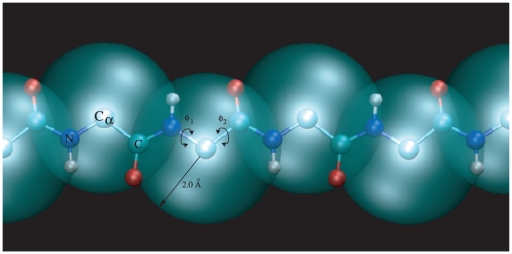
Illustration of the caterpillar model. The large transparent spheres represent the self-avoidance volume, which has a radius of 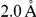, associated to an amino acid and centered on the position of the 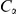 atoms. The backbone degrees of freedom are the torsional angles 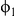 and 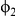. In order to describe hydrogen bonds also the backbone amide (NH) and the carboxyl (CO) groups are explicitly represented.

In order to support our hypothesis, we have developed a new model taking inspiration from the work of Maritan and co-workers, but unlike the tube model, the physico-chemical properties of individual amino acids are represented by an effective spherical potential centered on the 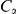 atoms, and a more realistic potential to represents the hydrogen bonding interactions. We refer to this model as the *caterpillar* model because of the image created by the spheres that follow the backbone (Fig. 1). The behavior of the caterpillar model depends on the balance between the spherical and hydrogen bond potentials. The main differences between the caterpillar and the tube model is that our model considers an arbitrary alphabet of amino acids and has a more detailed structure of the backbone that represents more faithfully the hydrogen bonding interactions. However, we retain the tube nature of the protein, via the self-avoiding core of the spheres centred on the 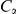 atoms [21]. We expect then the constraints resulting from the spherical and the hydrogen bonding potentials to confine the polypeptide chains, to regions of the conformational space with realistic protein-like structure elements. It is important to notice that the higher level of description of the caterpillar model allows not only for a higher precision in the representation of structures, but also to directly transfer the results obtained with the caterpillar model to the further refinement of full atomistic simulations. In fact, in order to further study the results of the caterpillar model with full atomistic simulations, we only need to add the atoms of the side chains of each amino acid directly on the backbone configurations of the coarse-grained simulations. Moreover, the use of spheres to account for self-avoidance is computationally more efficient [21] than the three-body interaction rules used in the tube model [18].

In this paper, we will show that the caterpillar model satisfies the two conditions mentioned above for foldabilty and designability, as it retains the elements of the polypeptide chain essential for the folding of designed sequences, and at the same time is simple enough to allow for an extensive exploration of the configurational space. Below we describe the novel design procedure based on the caterpillar model and we discuss the design of four representative protein structures taken directly from the Protein Data Bank (PDB) [22] [38]. We show that with our model we are able to design all test structures, and generate a large number of sequences with the target configurations stting at the bottom of a global free energy minimum. Finally, to further support the tangible link with real proteins we show that the hydrophobic/philic profile of designed sequences agrees with that typical of real sequences, and more importantly we demonstrate that the caterpillar model can refold the sequence of one of the four test proteins to its corresponding native structure.

## Methods

### Model

As outlined above, the caterpillar model is a 5-bead model with the 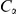 augmented by the full main atomic positions to introduce directional hydrogen bonds. The degrees of freedom of the model are the torsional angles 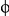 and 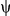; all other structural parameters are kept fixed at values from the literature [23]. The C, O, N, H positions were determined from the 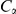 atoms as shown in Fig. 1.

The side chain interactions are represented by and effective 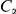-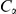 sphere-sphere interaction energy given by
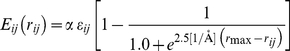
(1)where 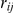 is the distance between the 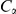 atoms at the centers of spheres 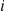 and 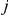 and 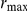 (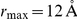) is the distance at which 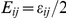; 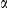 is a scale factor; see below. This expression provides a continuous square well form for the sphere-sphere interaction energy [39]. To determine the parameter 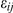 we made use of the model of Betancourt and Thirumalai (BT) [24], in which the interaction energies were derived from a calculation of the contact frequency in the PDB. This potential had been used primarily for lattice proteins, but it is also appropriate for the caterpillar model, which employs a square-well-like potential. Backbone hydrogen bonds were modeled with a 10–12 Lennard-Jones type potential using the expression [25]

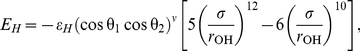
(2)where 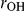 is the distance between the hydrogen atom of the amide group (NH) and the oxygen atom of the carboxyl group (CO) of the main chain. We set 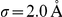, 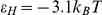, and 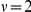; the values are given in [25].

To complete the parametrization, we need to determine 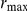 and 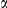. Since BT is a contact potential, there is no cutoff value. Here, we considered the 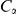-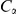 pair-correlation function g(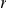) of several proteins and found that it begins to decay at approximately 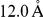 (see Figure S1). This behavior can be interpreted as the range of the effective interactions among amino acids. For larger values of 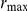, the system tends to acquire a mean field behavior, where every particle interacts with all the others, regardless of the geometry. By contrast, for smaller value of 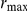, correlations that are crucial for the stability of the target structure can be missed. The parameter 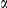 was chosen to balance the contributions of 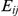 (Eq. (1)) and 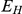 (Eq. (2)). With 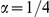, 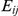 and 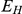 provide approximately the same contributions to the energy per particle. If 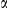 is too small, all sequences form 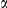-helices, while if it is too large all sequences fail to self-assemble and collapse in random glassy structures. In Eq. (2), the directionality of the hydrogen bonds is accounted for by multiplying the Lennard-Jones term by a factor containing the 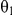 and 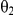 angles between the atoms COH and OHN, respectively. (Figures S2, S3 shows the distance dependence and angular dependence of 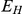 0). The directionality of the hydrogen bonds is essential to make more probable regions of conformational space characterized by the secondary structure elements typical of proteins. The spheres centered on the position of the 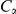 atoms ensure that only the maximum of the term in Eq. (2) for angles close to 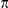 is accessible; that at 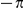 corresponds to configurations that are not allowed by the self-avoiding volumes of the spheres.

The energy function, Eq. (1), does not take the effects of the solvent into account explicitly. Although the designed sequences are able to fold to their respective target structures, their surface exposure profiles do not necessarily reproduce those of actual proteins. To improve this aspect of the design, we added an energy term 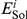 that penalizes the surfaces exposure of hydrophobic amino acids; the expression has the form 
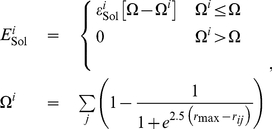
(3)where 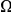 is a threshold for the number of contacts in the native structure above which the amino acid is considered to be fully buried and 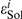 is the Dolittle hydrophobicity index [26], rescaled by 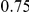 to make this term match the contributions from the other energy terms. The number of contacts for the amino acids in the native state varies between 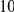 and 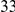; the value 24 was chosen for 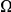 (see Fig. 5).

**Figure 2:**
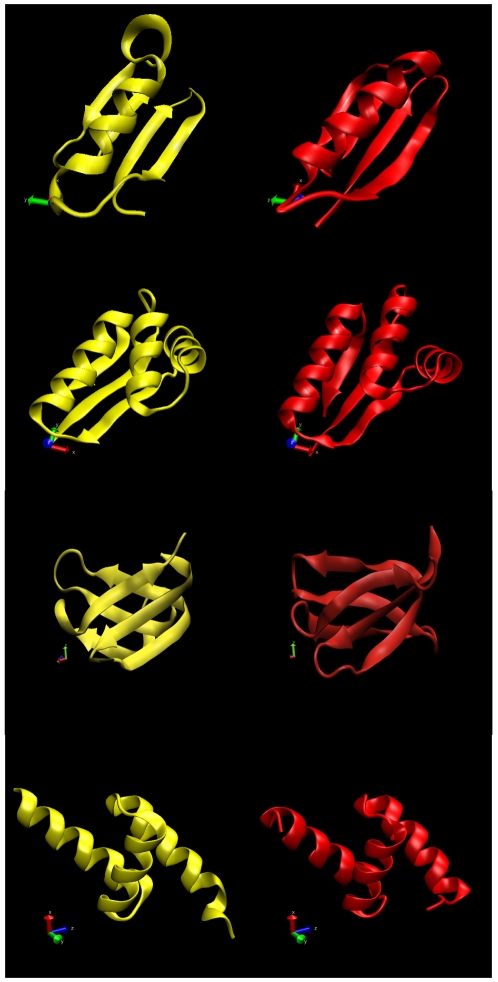
Comparison of the designed (yellow) and the target (red) structures for the four proteins analyzed in this work, from top to bottom. (a) Protein G (PDB ID 1PGB) 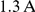 **DRMSD (** 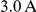 **RMSD 0); (b) L7/L12 (PDB ID 1CTF)** 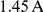 **DRMSD (** 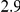 **RMSD 0); (c) lipoprotein (PDB id 2K57)** 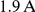 **DRMSD (** 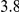 **RMSD 0); (d) UBA domain of Tap/NXF1 (PDB ID 1OAI)** 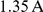 **DRMSD (** 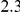 **RMSD 0).**

The designs described in this paper were done mainly using only Eqs. (1) and (2) for the energy. A comparison calculation was then made for one protein including the solvation energy term of Eq. (3).

### Design procedure

Given the potential function for the caterpillar model, there are two steps in the design procedure. First, a larger number (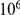) of sequences with a low energy and high sequence heterogeneity are generated using the target structure. Second, a selected subset is studied to determine its free energy surface and folding properties.

Several methods have been proposed to design the sequence of proteins such that they fold into a specific target conformation [27], [28], [13], [29]. We use here a modified version of a method that we described recently [13], which generates sequences by minimizing the energy of the target configuration and, at the same time, maximizes the number of amino acid permutations to increase the sequence heterogeneity. With this procedure the distribution of possible sequences remains large, which is necessary to generate sequences with a free energy minimum low enough to stabilize the folded state [27]. The search in sequence space is carried out by a parallel tempering Monte Carlo procedure with single point mutation moves. As in the conventional Metropolis scheme, the acceptance of trial moves depends on the ratio of the Boltzmann weights at a design temperature 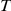 of the new and old states [30]. However, if this were the only criterion, there would be a tendency to generate homopolymer chains with a low energy, rather than chains that fold selectively into a specific target structure. To ensure an amino acid composition far from the homopolymer region of the sequence space, we impose the following acceptance criterion for a single mutation 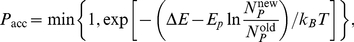
(4)where 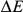 is the difference of the energy before and after the mutation attempt, 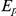 is a scale factor for the relative value of the two terms in the equation, and 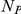 is the number of permutations that are possible for a given set of amino acids; 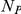 is given by the multinomial distribution 
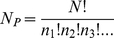
(5)where 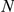 is the total number of monomers and 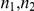, etc are the number of amino acids of type 1,2, etc. While sampling the sequence space with the Monte Carlo scheme, we set 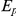 to high enough value 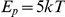 to generate sequences with a heterogeneous composition. To adequately sample the sequence space, we generated 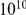 sequences with the native structure as the template using the parallel tempering scheme [13] with a set of temperatures 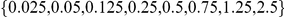 in units of 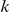. From these we selected the ones most likely to yield stable structures for the native state. For this purpose, we used the Landau free energy 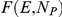, defined by
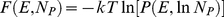
(6)and generate the two-dimensional normalized histogram 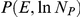 of the distribution of the pair (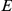 and 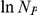) collected over the ensemble of the 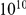 generated sequences. For further study we chose a small number of sequences with low Landau free energy; i.e., ensembles of sequences that have a reasonably low energy and a high probability of being observed. The rationale for this choice is that such sequences are robust against point mutations, which are correlated with the overall thermodynamic stability ([31], [32]; see also [33]). Our criterion can be understood with a simple argument in the mean-field approximation, where we consider only short range correlations between the amino acids in the chains. In these conditions point mutations are equivalent to small structural distortions, as both perturbations only have a local effect. Hence, proteins that are robust against point mutations are most probably resistant to small deformations induced by thermal fluctuations.

For each selected sequence, we computed the free energy 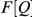 as a function of a the order parameter 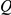, where 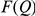 is defined by 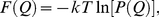
(7)where 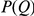 denotes a normalized histogram of the number of sampled conformations with order parameter 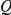, and 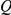 is the Distance Root mean square difference (DRMSD) from the native structure. In practice, a direct calculation of this histogram is not efficient, since even the caterpillar model tends to be trapped in local minima, especially at low temperatures. To induce escape from these local minima, we made use of the Virtual Move Parallel Tempering Monte Carlo sampling scheme proposed by Coluzza and Frenkel [34], based on the Waste Recycling approach [35]. This scheme is very efficient in sampling both high and low free energy states (see supplementary informations). We find that on a 4 quad-core dual Xeon (Harpertown) compute nodes the calculation of 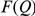 as a function of 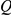 for a single sequence requires 336 hours of CPU time, while generation of the 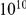 sequences requires only 2 hours CPU time.

We used the native conformations of four representative proteins as target structures (see Fig. 2), the B1 immunoglobulin-binding domain of streptococcal protein G (PDB ID 1PGB), the C-terminal domain of the ribosomal protein L7/L12 of E. coli (PDB ID 1CTF), a putative lipoprotein from *Pseudomonas syringae* (Gene Locus PSPTO2350, PDB code 2K57), and the UBA domain of Tap/NXF1 (PDB ID 1OAI).

**Figure 3:**
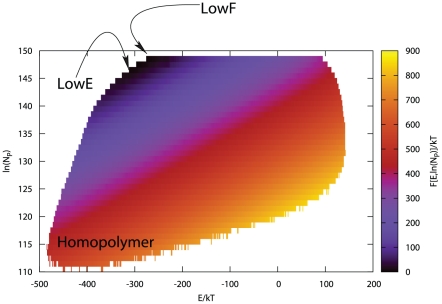
Plot of the design free energy surface 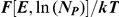 for protein L7/L12 (PDB ID 1CTF) as a function of the total 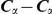 energy and the logarithm of the number of possible letter permutations 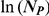. For small values of 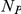 the sequences will tend to be more and more homopolymeric. The most stable sequences corresponds to the to lowest free energy point (indicated by the the LowF arrow) and the folding capacity deteriorates moving away from that point even if the total energy is lower (e.g. the point indicated by the LowE arrow). The boundaries are determined by the limits in the computational power but also by the fact that some combinations of 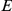 and 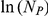 are not possible.

## Results

**Figure 4:**
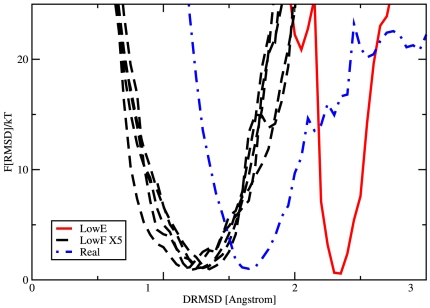
Comparison of the folding free energies 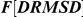 of 6 designed sequences and of the real sequence for L7/L12 as a function of the root mean square distance (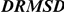) from the target structure. The profile of 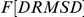 (black dashed line) for 5 sequences selected from the ensemble of those with the lowest free energy in sequence space (LowF in Fig. 3) is compared with the profile (red line) obtained for a sequence with lower energy (LowE) than the previous ones. The free energy has been calculated at the same temperature 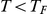. The folding efficiency of the LowF sequences is very different from the one of LowE as the latest one cannot reach a proper folded structure. Finally we also plot the folding free energy for the real sequence (Real) of the same protein L7/L12 (point dash blue line). At 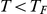, we found the minimum of 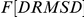 to be around 1.6 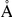 (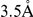 RMSD), indicating that the designed proteins are folded correctly on their targets.

The Landau free energy diagram 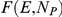 for protein 1CTF, which we studied in detail, is shown in Fig. 3. As is evident from the diagram, the lowest energy sequences and lowest Landau free energy are not directly correlated; i.e., there are numerous very low energy structures with sequences that have a low probability of being observed. We then calculated the free energy as a function of the DRMSD from the native structure (Eq. (7)) for five selected low free energy, high heterogeneity sequences of protein 1CTF; they corresponds to the point indicated by the arrow labeled “LowF” in Fig. 3. Figure 4 shows the free energy surfaces for these proteins at a low temperature where the proteins are stable with the present energy function, it is a relatively smooth surface with the minima of the free energy at an DRMSD in the range 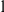 to 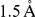; the breadth of the surface can be argued to reflect the structural fluctuations present in the native state. Because of the definition of DRMSD, structures that are long lived would appear as free energy minima at high values of DRMSD. Hence, the smoothness of the free energy profiles in Fig. 4 indicates that the folding process of our artificial sequences occurs spontaneously with no long lived metastable states. An important result is that in the low temperature simulations, the free energy surface shows no misfolded states with free energies below that of the target structure. It is important to notice that in order to have a single free energy minimum, we did not explicitly impose to the design process to disfavor particular conformations of the chain. Similar results for the other three systems are given in supplementary informations (Figure S4), and overall we get a structure prediction precision between 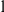 and 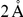 in DRMSD (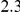 and 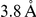 in RMSD [40]) as shown in Fig. 2.

**Figure 5:**
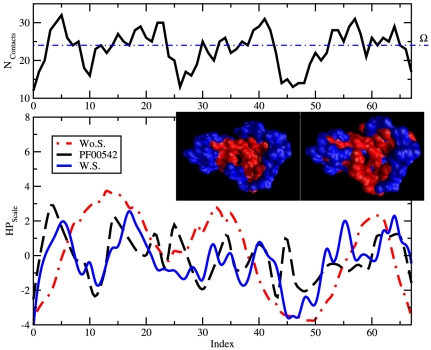
Hydrophobic/philic profile of the protein L7/L12 (PDB ID 1CTF) designed with and without the solvation term. In the top frame we plot the number of contacts that each amino acids along the chain has with the all the other non consecutive amino acids in the range of 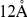 defined by our potential in Eq. (2). Large numbers indicate amino acids that are buried in the core of the protein while low number correspond to residues that are highly solvated. The dashed horizontal line refers to the value 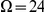 in Eq. (2). In the bottom frame we compare the hydrophobic/philic profiles averaged over the designed sequences, with (W.S., blue continuous line) and without (Wo.S., red point-dash line) the solvation term in Eq. (3), to the average profile obtained from the Pfam alignment data (PF00542, black dashed line) corresponding to the structure L7/L12. W.S. sequences capture many of the features of the HP profiles of the PF00542 and follows more closely the profile described in the top frame, indicating that we design proteins with an hydrophobic core surrounded by hydrophilic amino acids, which overall is more realistic. It has to be noted that the discrepancies between the designed and the real proteins (between residue 20 and 30 and around residue 45) occur in regions where structurally one would expect hydrophilic amino acids. The unexpected hydrophobic patches present in the wild type proteins may very well be involved in the function of the protein in vivo that we do not take into account during the design procedure. In the inset From left to right, comparison of the designed (W.S.) and the native hydrophilic (blue) and hydrophobic (red) amino acids distributions for L7/L12.

We tested if the sequence selection mechanisms, based on the Landau free energy, performed better than simply taking a low energy sequence, as was done previously for lattice proteins [27]. Figure 4 also shows the results of a sequence selected for its low energy (LowE) with a relatively high number of permutations; see Fig. 3. In order to show how important is to select sequences from the most probable ensemble, we chose the LowE sequence not too far from the global sequence free energy minimum. Nevertheless, the folding of “LowE” is significantly less reliable than that of the LowF sequences, as the equilibrium configuration of LowE draomatically differs from the native structure.

We finally introduced the solvation term in equation (3). By including the latter we repeat the design procedure for L7/L12, and the refolding for the natural sequence of L7/L12 as taken from the PDB (1CTF). We set 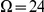. In figure 5 we plot the hydrophobic/philic profile (HP) of the protein 1CTF designed with and without the solvation term in Eq. (3). The first important observation is that even with our 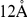 ranged potential (Eq. (2)), we are able to distinguish between buried amino acids and surface residues, as is demonstrated by the large variation in the number of contacts (top frame). Moreover the HP profiles averaged over the designed sequences with solvation term (W.S.) follow much better the contact profile than the profile relative to sequences designed without the solvent term (Wo.S.), indicating that our “artificial” proteins have a hydrophobic core surrounded by hydrophilic amino acids as expected for molecules that live in aqueous solutions [36]. Finally we compared the artificial HP profiles to the average profile obtained from the Pfam alignment data (PF00542) for protein 1CTF; the curve for W.S. sequences is qualitatively comparable to one of the real proteins, as the discrepancies (between residue 20 and 30 and around residue 45) occur in regions where the wild type proteins express hydrophobic residues even if highly exposed to the solvent, which could be the results of functionalities that we did not include in the design procedure. At this point it is natural to ask if the caterpillar model, with the solvation term, is able to reproduce the folded structures of real proteins, since we have shown that designed sequences refold to the target structure, and the design now produces protein like sequences. In Fig. 4 we plot the folding free energy profile of the natural sequence of protein L7/L12. The profile is qualitatively similar to the one obtained from the folding of the artificial sequences, and the distance of the global free energy minimum from the X-Ray structure is still small (1.6 Å DRMSD, 3.4 Å RMSD). Hence, the quality is again striking considering that the only parameters we had to adjust in the model are the range of the potential, the scaling factor of the 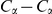 interaction and the threshold 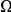 of the solvation term.

We conclude that a carefully tuned external field can produce a protein-like hydrophobicity profile (Fig. 5) and closely predict the native structure of the real sequence (Fig. 4). The proposed framework is then fully self consistent since the design procedure is able to produce natural-like sequences, while the folding properties of the caterpillar are compatible with the folding of real natural sequences. Moreover, the estimation of all free parameters is based on the condition that designed sequences must refold into their respective target structure, and as a result we reattain fundamental properties of real proteins that we did not impose to the system. Model and methodology are therefore shown to be an important step forward in bridging the crucial gap between a coarse grained representation and a fully atomistic description of proteins.

## Discussion

In this work we introduce a fundamental criterion for the designability of coarse-grained models of proteins. With the caterpillar model we are able to design protein sequences for various proteins representative of the typical combinations of protein secondary structures. Each of the tested sequences reached the target structure with a very high precision considering the simplicity of the model, demonstrating that the procedure is universal for proteins with different proportions of alpha helices and beta sheets. With our model we could characterize in detail the free energy of the folding process, and we showed that each of the free energy landscapes has a global free energy minimum near the target structures. Moreover, the landscapes are relatively smooth indicating that our designed proteins can spontaneously fold without remaining trapped for long time in metastable states.

The caterpillar model provides a strong evidence to support our hypothesis that a minimum number of constraints is necessary in order to successfully perform protein design. By applying an accurate representation of the backbone we demonstrated that design and folding of real proteins is possible to a degree of accuracy that could not have been anticipated given the level of coarse-graining applied. To the best of our knowledge, a direct analysis of the importance of constraints for the design of protein like structures has never been done before. Our results, then not only extend protein design beyond lattice proteins but also further extend the important work of Maritan and co-workers [17], [18], [19] on the tube model. With the tube model, the authors showed that the protein structure universe is largely determined by the particular geometry imposed by the backbone, independently of the accuracy used to represent the amino acid pair interactions. With the caterpillar model we not only verify the results of Maritan and co-workers, but also we extend the function of the backbone geometry to the crucial role of enforcing the minimal set of constraints responsible for the protein design property.

It is important to stress that the three free parameters of the model have been adjusted only on the refolding ability of the designed sequences, and, as a result, the artificial sequences resemble real proteins in the hydrophilic/phobic profiles, and the folding of real sequences predicts the correct native structure with a surprising high accuracy. This last result suggests that it is possible to determine a universal set of values for the parameters valid for all proteins, which we intend to make the center of further investigation. Moreover, given its computational efficiency, we anticipate that the caterpillar model will be useful for studying other important aspects of protein behaviour such as folding, misfolding and aggregation. Especially considering that, thanks to the high detail of the backbone, the results of our model can be easily integrated in full atomistic simulations by adding the side chains of each amino acid.

## Supporting Information

Figure S1

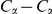
 Radial distribution function 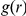 of three of the target proteins tested in our work. The solid lines are spline interpolations of the data points to guide the eye. The plots show common features between all three proteins, in particular the position of the major peaks is contained in the 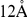 radial distance. This alone is not enough to prove that the effective potential between 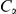 pairs should have such a wide range, but it supports our phenomenological observation that shorter or longer ranges do not guarantee the same universal refolding properties to the caterpillar model.(EPS)

Figure S2 Angular dependence of the potential used to model hydrogen bonds in Eq.(2).
(EPS)

Figure S3 Radial dependence of the potential used to model hydrogen bonds in Eq.(2).
(EPS)

Figure S4Free energies 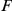
(DRMSD) of the designed sequences as a function of the root mean square distance (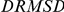) from their target structures for the four cases that we considered in this work: (a) the B1 immunoglobulin-binding domain of streptococcal protein G (PDB ID 1PGB), (b) the C-terminal domain of the ribosomal protein, (c) a putative lipoprotein from *Pseudomonas syringae* (Gene Locus PSPTO2350, PDB code 2K57), and (d) the UBA domain of Tap/NXF1 (PDB ID 1OAI). The free energy is shown for two temperatures, the first (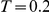) slightly below the folding temperature (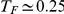) and the second (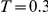) slightly above; all temperatures are in reduced units). At low temperatures, for all the target structures that we considered we found the minima of 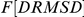 to be between 1.0 and 1.5 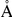, indicating that the designed proteins are folded correctly on their targets. At 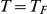 the native is at equilibrium with the unfolded state. The exact determination of the folding temperature requires a fine analysis of the temperature dependence of the folding process, and is beyond the scope of our work. Our estimate is based on the observation that just above 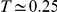 the protein is unfolded, while below the native state is the most stable state.(EPS)

Text S1 Supporting information.
(PDF)
