# Supplementary material for: A Coarse-Grained Approach to Protein Design: Learning from Design to Understand Folding
Source: PLoS One. 2011 Jul 1;6(7):e20853. doi: 10.1371/journal.pone.0020853 (PMC3128589; doi:10.1371/journal.pone.0020853)
Supplement: Text S1 — Supporting information. (PDF) [file pone.0020853.s005.pdf]

Supporting informations for
A coarse-grained approach to protein design: learning from design to understand folding

Ivan Coluzza[1]

[1]*Department of Physics, University of Vienna, Boltzmanngasse 5, 1090 Vienna, A.*

Virtual-Move Parallel Tempering

The VMPT scheme is a combination of the adaptive parallel tempering algorithm [1] and the waste recycling method developed by Frenkel [2]. This method consists in using the information about the reject moves to generate a bias potential that helps the system visit regions of the phase space that are otherwise difficult to reach. In the conventional MCMC method all information about rejected trial moves is discarded. Recently Frenkel has proposed a scheme that makes it possible to include the contributions of rejected configurations in the sampling of averages [2]. In the standard parallel tempering (PT) scheme, we only retain information about PT moves that have been accepted. However, in the spirit of Ref. [2], we can include the contribution of all PT trial moves, irrespective of whether they are accepted. The weight of the contribution of such a virtual move is directly related to its acceptance probability. For instance, if we use the symmetric acceptance rule for MC trial moves, then the weights of the original and new (trial) state in the sampling of virtual moves are given by

$$\begin{aligned} P_N &= \frac{e^{\Delta\beta\Delta E_{O\to N}+\Delta W_{O\to N}}}{1+e^{\Delta\beta\Delta E_{O\to N}+\Delta W_{O\to N}}} \\ P_O &= \frac{1}{1+e^{\Delta\beta\Delta E_{O\to N}+\Delta W_{O\to N}}}\,, \end{aligned}$$

where W is a bias potential. We are not limited to a single trial swap of state i with a given state j. Rather, we can include all possible trial swaps between the temperature state i and all $N-1$ remaining temperatures. Our estimate for the contribution to the probability distribution P_i corresponding to temperature i is then given by the following sum

$$\begin{aligned} P_i(Q) &= \sum_{j=1}^{N-1}\left(\frac{1}{1+e^{\Delta\beta_{ij}\Delta E_{ij}+\Delta W_{ij}}}\right)\delta(Q_i-Q)+ \\ &\quad \sum_{j=1}^{N-1}\left(\frac{e^{\Delta\beta_{ij}\Delta E_{ij}+\Delta W_{ij}}}{1+e^{\Delta\beta_{ij}\Delta E_{ij}+\Delta W_{ij}}}\right)\delta\left(Q_j-Q\right), \end{aligned}$$

where the delta functions select the configurations with order parameter Q. The combination with the parallel tempering is particularly efficiently because the information about the sampled states is shared between all the simulations running at different temperatures, that naturally will tend to explore different regions of the phase space. In the case of proteins it means that at low temperature it is possible to know what bias is needed to reach the unfolded state because that is sampled more often at higher temperatures.

[1] Faller R, Yan Q, de Pablo J (2002) Multicanonical parallel tempering. J Chem Phys 116: 5419–5423.

[2] Frenkel D (2004) Speed-up of monte carlo simulations by sampling of rejected states. P Natl Acad Sci USA 101: 17571–17575.
